# Shade trees preserve avian insectivore biodiversity on coffee farms in a warming climate

**DOI:** 10.1002/ece3.6879

**Published:** 2020-10-23

**Authors:** Sarah L. Schooler, Matthew D. Johnson, Peter Njoroge, William T. Bean

**Affiliations:** ^1^ Wildlife Department Humboldt State University Arcata CA USA; ^2^ Department of Environmental and Forest Biology State University of New York School of Environmental Science and Forestry Syracuse NY USA; ^3^ Ornithology Section National Museums of Kenya Nairobi Kenya; ^4^ Biology Department California Polytechnic State University – San Luis Obispo San Luis Obispo CA USA

**Keywords:** bird community, climate change, coffee, East Africa, Kenya, Maxent, shade coffee

## Abstract

**Aim:**

Coffee is an important export for many developing countries, with a global annual trade value of $100 billion, but it is threatened by a warming climate. Shade trees may mitigate the effects of climate change through temperature regulation that can aid in coffee growth, slow pest reproduction, and sustain avian insectivore diversity. The impact of shade on bird diversity and microclimate on coffee farms has been studied extensively in the Neotropics, but there is a dearth of research in the Paleotropics.

**Location:**

East Africa.

**Methods:**

We created current and future regional Maxent models for avian insectivores in East Africa using Worldclim temperature data and observations from the Global Biodiversity Information Database. We then adjusted current and future bioclimatic layers based on mean differences in temperature between shade and sun coffee farms and projected the models using these adjusted layers to predict the impact of shade tree removal on climatic suitability for avian insectivores.

**Results:**

Existing Worldclim temperature layers more closely matched temperatures under shade trees than temperatures in the open. Removal of shade trees, through warmer temperatures alone, would result in reduction of avian insectivore species by over 25%, a loss equivalent to 50 years of climate change under the most optimistic emissions scenario. Under the most extreme climate scenario and removal of shade trees, insectivore richness is projected to decline from a mean of 38 to fewer than 8 avian insectivore species.

**Main conclusions:**

We found that shade trees on coffee farms already provide important cooler microclimates for avian insectivores. Future temperatures will become a regionally limiting factor for bird distribution in East Africa, which could negatively impact control of coffee pests, but the effect of climate change can be potentially mediated through planting and maintaining shade trees on coffee farms.

## Graphical abstract

Shade trees may mitigate the effects of climate change through temperature regulation that can aid in coffee growth, slow pest reproduction, and sustain pest‐eating bird diversity. We created current and future regional Maxent models for avian insectivores in East Africa and then adjusted current and future bioclimatic layers based on mean differences in temperature between shade and sun coffee farms, and projected the models using these adjusted layers to predict the impact of shade tree removal on climatic suitability for avian insectivores. We found that future temperatures will become a regionally limiting factor for bird distribution in East Africa, which could negatively impact control of coffee pests, but the effect of climate change can be potentially mediated through planting and maintaining shade trees on coffee farms.

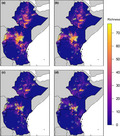

## 1 INTRODUCTION

Coffee is a crucial source of income for many developing countries, with 25 million people, largely smallholders, depending on its production for their livelihoods (Avelino et al., 2015; Bunn et al., 2015). Additionally, coffee is one of the most important global crops, being one of the most heavily traded global agricultural commodities and a highly popular beverage consumed by approximately one‐third of the world's population (DaMatta et al., 2019; Donald, 2004; Vega et al., 2003). However, climate change is predicted to decrease global suitability for coffee growth by as much as 50% before 2050 (Bunn, et al., 2015; Moritz & Agudo, 2013; Pham et al., 2019; Rahn, et al., 2018). Climate change is expected to impact coffee production directly (e.g., through physiological response of coffee plants) and indirectly (e.g., through changing pest regimes; Bunn, et al., 2015; Jaramillo et al., 2011, 2013; Pham et al., 2019). Direct impacts are generally expected to be negative due to temperature sensitivity of the plants (Bunn, et al., 2015; Magrach & Ghazoul, 2015; Rahn, et al., 2018), although increased atmospheric CO_2_ may mitigate these impacts through increased carbon fertilization (DaMatta et al., 2019; Rahn, et al., 2018; Verburg et al., 2019). However, a growing body of evidence indicates that increasing temperatures will negatively impact coffee by increasing pest fecundity (Bale et al., 2002; Jaramillo et al., 2011; Magrach & Ghazoul, 2015). It has been hypothesized that shade trees may mitigate the effects of a warming climate by lowering temperature and increasing humidity on coffee farms (Jha et al., 2014; Pham et al., 2019; Rahn, et al., 2018).

Coffee has been traditionally grown under a diverse canopy of native shade trees, but as management for higher short‐term yields has intensified, the use of shade trees has decreased (Jha et al., 2014). Yet, the supposition that coffee grown without shade (sun coffee) provides higher quantity yields than shade coffee is unproven; in fact, recent research has reported that up to 50% shade cover on farms has a positive effect on coffee yields both in quantity and quality due to temperature regulation, shade tree nitrogen fixation, reduction of coffee related pests, and decreased spread of coffee‐effecting diseases (Atallah et al., 2016; Avelino et al., 2015; Cerda et al., 2017; Coffee Research Institute, 2019; Jha et al., 2014; Jonsson et al., 2015; Maas et al., 2013, 2016; Meylan et al., 2017; Soto‐Pinto et al., 2000). Shade negatively impacts coffee pests by lowering temperatures below pests’ thermal optima and also contributes to pest control through increased predation by birds (Johnson et al., 2010; Kariuki Ndang'ang'a et al., 2013; Karp et al., 2013; Kellermann et al., 2008; Maas et al., 2016; Mäntylä et al., 2011; Perfecto et al., 1996, 2014; Railsback & Johnson, 2014). Studies in the Neotropics have shown that increased predation may be a function of overall bird abundance or diversity (Van Bael et al., 2008; Martínez‐Salinas et al., 2016; Sekercioglu, 2012). Though less well‐studied in the Paleotropics, it follows that greater avian insectivore diversity and/or abundance in shade coffee in the Paleotropics may also contribute to pest control (Buechley et al., 2015; Chain‐Guadarrama et al., 2019; Dainese et al., 2019; Milligan et al., 2016).

Eastern Africa is one of the few locations in the world projected to become more suitable for growing coffee in the future, with parts of current growing regions remaining climatically suitable and availability of future climatically suitable area upslope of current production (Bunn, et al., 2015; Bunn et al., 2015; Davis et al., 2012; Ovalle‐Rivera et al., 2015). With an estimated 20% of the world's 10 million ha of coffee, more research is needed in the region to predict the impacts of climate change on coffee (Global Commodity Production Statistics, 2016). Previous empirical work has documented the distribution of coffee pests (Jaramillo et al., 2011), current and future coffee distribution (Bunn, et al., 2015; Rahn, et al., 2018), and effects of shade on coffee production in East Africa (Ziska et al., 2018). However, there have been no studies on the current and future distribution of insectivorous bird species in East Africa and how that distribution may change in relation to microclimate on shaded coffee farms. Like in the Neotropics, shade coffee farms in East Africa exist on a gradient from small, heterogeneous farms that can support a high diversity of shade trees (Buechley et al., 2015), to large‐scale sun coffee plantations with few to no shade trees (Moguel & Toledo, 1999). Large partially shaded monocultures are common and marked by a low density and low diversity of shade trees, with the community primarily consisting of *Grevillea robusta*, *Cordia africana,* and *Albizia sp*., as they grow rapidly, are leguminous and thus fix nitrogen, and are relatively easy to maintain (Jha et al., 2019; Liebig et al., 2016; Rahn, et al., 2018, Schooler unpublished data). While the presence of certification schemes (e.g., Bird Friendly^®^ Rainforest Alliance^®^) is less conspicuous in East Africa than in the Neotropics, the use of shade trees on coffee farms in East Africa is increasing from education efforts (Coffee Research Institute, 2019; Liebig et al., 2016, Schooler, unpublished data).

Species distribution models are a valuable tool for determining current and future regional distributions of species based on climate, but have limited ability to include the effects of local microclimate factors, such as shade (Araujo & Pererson, 2012; De Frenne *et al*., 2013). Shade strongly affects temperature and humidity on coffee farms and thus may decrease a regional model's ability to predict local scale climatic suitability (Evans et al., 2016; Garedew et al., 2017; Rapacciuolo et al., 2014). On a local scale, shade trees may mediate current and future temperature and humidity extremes, preserving suitability for coffee and birds even within larger areas expected to suffer effects of climate change (Buechley et al., 2015; Pearson & Dawson, 2003). Planting and maintaining shade trees on coffee farms is one of the few management actions available to landowners to mitigate the impacts of climate change (Hirons et al., 2018; Ziska et al., 2018). Due to shade's importance for both coffee growth, pest reproduction, and bird abundance and diversity, it is important to determine the impacts that shade will have on current and future insectivorous bird distributions. Understanding this relationship will be crucial in the future as temperature extremes become more common (Anwar et al., 2013).

In this study, we modeled regional current climatic suitability for a wide variety of avian insectivores that may contribute to pest control on coffee farms in East Africa. Temperatures from shade and sun coffee plantations in South Central Kenya were used to estimate the microclimatic effects of shade trees on coffee farms. Finally, this study projected the regional suitability models into the future, focusing particularly on areas predicted to be suitable for coffee. We compared models with and without the temperature mitigating effects of shade trees in order to evaluate the relationships between birds, shade, and coffee.

## 2 METHODS

### 2.1 Study area

To assess large‐scale insectivorous bird climatic suitability, we examined bird species distributions across northeast Africa including Ethiopia, Kenya, Tanzania, Uganda, Burundi, and Rwanda. In this region, coffee is generally cultivated in the highlands between 1,300 and 2,200 meters above sea level (Bunn, et al., 2015; Global Commodity Production Statistics, 2016). The geography of East Africa ranges from the savannah of southern Kenya to the rainforests of Uganda. Mean annual rainfall varies from 400 mm to over 2,500 mm. The region is broadly characterized by two distinct wet seasons, one between March and May (“long rains”) and the other during October and November (“short rains”).

### 2.2 Temperature data collection

**Figure 1:**
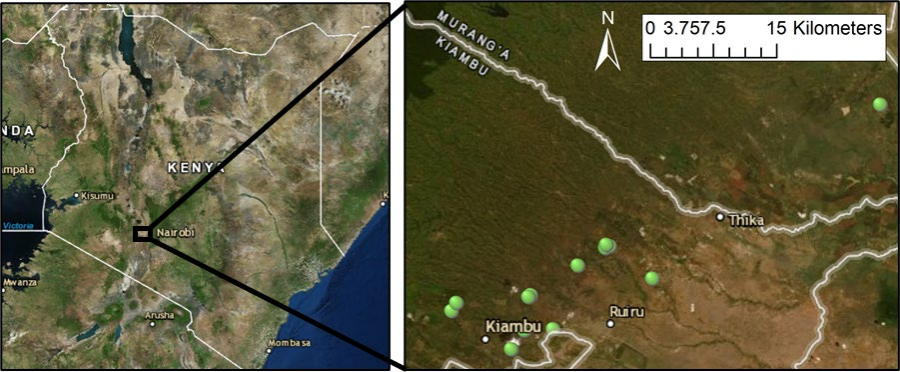
Locations where iButtons were placed (*n* = 13) on coffee farms marked with points in Kiambu and Muranga Counties shown as location within Kenya. Large towns and county lines are labeled for reference

To assess the impact of shade trees on microclimate, we collected temperature data from 13 coffee sites with varying shade levels (selected opportunistically) in Kiambu and Muranga counties in Kenya across an elevation gradient from 1,450 to 1950 meters (Figure 1). Kiambu and Muranga counties are ~2,500 and 2,325 km^2^, respectively, and are located north and east of Nairobi, Kenya between latitude 1°14′52″ to 0°56′83″S and longitude 36°39′52″ to 37°41′79″E. Temperature logger locations on coffee sites were randomly generated at least 50 m from the site edge. If shade trees were located within 50 m of the randomly generated point on the site, a second temperature logger was placed underneath the closest shade tree, therefore some sites had multiple loggers (*n* = 4 sites had two loggers; total of *n* = 13 sites; Figure 1).

We placed temperature loggers in coffee bushes beneath shade trees (*n* = 7 data loggers) and in full sun (*n* = 10 loggers). Maxim iButton^©^ temperature loggers (“iButtons”) were mounted using 3M Command^©^ strips on 2 mm thick white plastic cut ~3 by 4 cm squares with a 1 mm diameter hole drilled at the top. Loggers were then attached to coffee bush trunks below coffee bush tops shaded from direct sunlight averaging 2 m above ground using zip ties (Garedew et al., 2017). iButtons were set to collect data once per hour and collected data for approximately three months (until March 2018). All fieldwork complied with Humboldt State University Institutional Animal Use and Care Committee guidelines (protocol number 16/17.W.06‐A).

### 2.3 Species selection and data use

**Figure 2:**
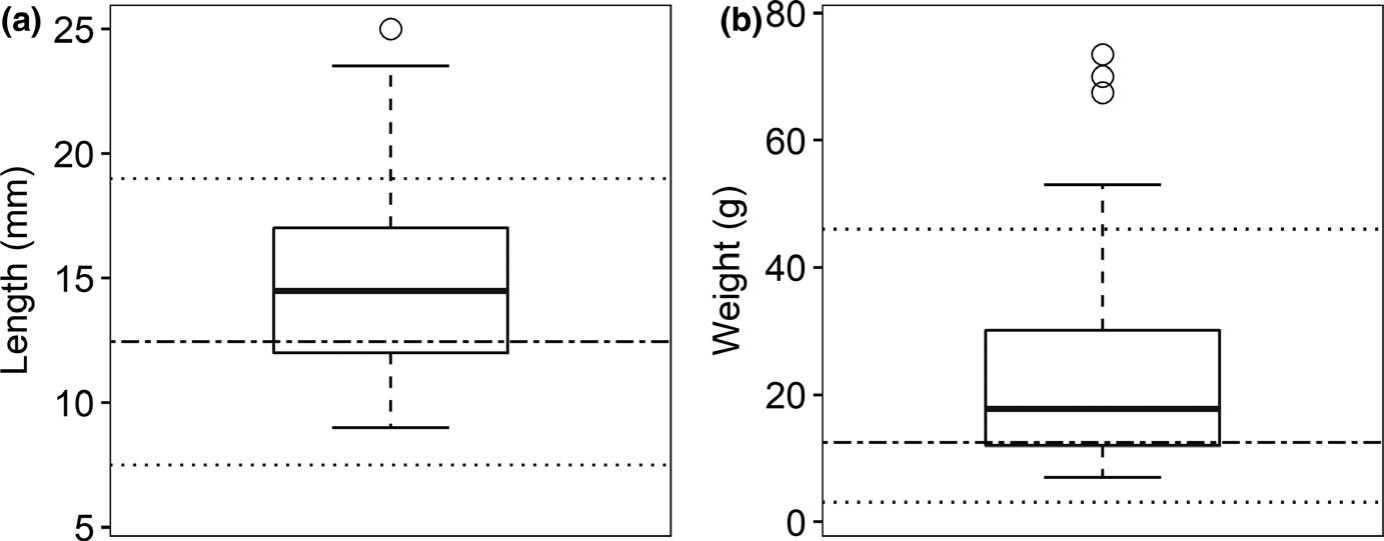
Box plot of length (a) and weight (b) of bird species included in this study (*n* = 77). Dashed lines show maximum and minimum lengths and weights of bird species found to eat coffee berry borer in the Neotropics, and dot‐dashed lines indicate mean lengths and weights of bird species found to eat coffee berry borer in the Neotropics (Table 1) (*n* = 12) (Karp et al., 2014; Martínez‐Salinas et al., 2016; Sherry et al., 2016)

To determine which bird species to model in East Africa, we used species recorded by Smith et al. (2015) from mist‐net surveys conducted December 2012–January 2013 and December 2013–January 2014 (when migratory species were present) in Nyeri County, Kenya and by Schooler (2019) from point‐count surveys conducted December 2017–January 2018 in Kiambu and Muranga counties, Kenya. Potential pest‐consuming species were selected from this pool (*n* = 203) through diet, length, and weight (del Hoyo et al., 2018). Diet classifications included omnivores and insectivores, and bird measurements were used to identify species similar to those known to eat coffee pests in the Neotropics (Table 1; Johnson et al., 2010; Karp et al., 2014; Martínez‐Salinas et al., 2016; Sherry et al., 2016). We included any insectivorous or omnivorous bird species with overall lengths ≤25 cm and weights ≤73.5 g. These values exceeded the maximum lengths and weights of Neotropical bird species found to eat coffee pests by 6 cm and 27.5 g, respectively, (Table 1, Figure 2) to better encompass potential natural enemies of insect pests within the bird composition occurring in East Africa. The final list included 77 bird species from 20 families, with a length range from 9 to 25 cm (mean = 14.91) and a weight range from 7 g to 73.5 g (mean = 23.13; Table 1, Figure 2, Table S1).

**Table 1:** Lengths and weights of neotropical birds determined to eat coffee pests through gastric lavage or guano analysis with location of study

Species	Location	Length (cm)		Weight (g)	
		Min	Max	Min	Max
American Redstart^1^	Jamaica	11	13	6.5	12
Black‐and‐white Warbler^1^	Jamaica	11	13	8.8	15.2
Black‐throated Blue Warbler^1^	Jamaica	12	14	8.4	12.4
Northern Parula^1^	Jamaica	10.5	12	7.1	10.2
Prairie Warbler^1^	Jamaica	11	12	5.7	10.8
Alder Flycatcher^2^	Costa Rica	13	17	12	14
House Wren^2^	Costa Rica	11.5	12.5	8.9	14.2
Common Tody‐flycatcher^2^	Costa Rica	8.8	10.2	4.4	8
Buff‐throated Foliage‐gleaner^3^	Costa Rica	18	19	30	46
Rufous‐breasted Wren^3^	Costa Rica	14	14	13.5	18.5
Rufous‐capped Warbler^3^	Costa Rica	13	13	7	16
White‐tailed Emerald^3^	Costa Rica	7.5	8	3.1	3.3

^1^ Sherry et al. (2016).

^2^ Martínez‐Salinas et al. (2016).

^3^ Karp et al. (2014).

Following bird species selection, all occurrences in Ethiopia, Kenya, Tanzania, Uganda, Burundi, and Rwanda collected after 1970 for each bird species were downloaded from the Global Biodiversity Information Facility (GBIF; GBIF.org, 2018). Background points (“available points,” “pseudoabsence points”) for use in species distribution models were generated randomly throughout East Africa (Ethiopia, Kenya, Tanzania, Uganda, Burundi, and Rwanda). The number of background points generated was the same as the number of observed points for each species (Table S2). Current (1970 – 2000 average) and future climate data were downloaded from WorldClim bioclimatic variables (“Bioclim”) using the dismo package in R (Fick & Hijmans, 2017). For future climate projection, we analyzed both the most conservative and the most extreme climate scenarios of 2.6 and 8.5 representative concentration pathways (RCPs), respectively, projected for 2075. We chose to use the climate model HadGEM2‐AO because it has been shown to be an accurate future climate predictor model for East Africa (Onyutha et al., 2016). We included limited results for the 2.6 representative concentration pathway with full results in the appendix and expanded on results for the 8.5 representative concentration pathway to determine results for the most extreme scenario of climate change.

### 2.4 Maxent modeling

Research modeling current and future species distributions relies upon species distribution models, with Maxent (Maximum Entropy Modeling) among the most commonly used methods (Phillips et al., 2017; Warren & Seifert, 2011; Yalcin & Leroux, 2017). Species distribution models (also called environmental or ecological niche models, habitat suitability models, correlative distribution models, or climatic envelope models) use environmental data such as climate to predict regional suitability for a given species. The resulting maps are often the basis for estimated species distributions. The theoretical underpinning for these models is that climate is an underlying factor in all coarse‐scale species distributions, so climatic factors can serve as a proxy for more complex biotic and abiotic interactions and thus can functionally predict species distributions on a regional scale (Guisan & Thuiller, 2005). Over a large community of species, it is not feasible to measure specific biological impacts such as competition or predation, so species distribution models are especially useful in the face of climate change (Pearson & Dawson, 2003). We chose Maxent because is easy to use, gives reliable extrapolation, and requires less computing power than other methods (Benito et al., 2013; Elith et al., 2011).

To minimize the risk of overfitting and aid in interpretation of model results, we selected nine out of nineteen possible Bioclim predictors based on biological underpinnings and interpretability. The predictors used were as follows: annual mean temperature, mean diurnal range, maximum temperature in the warmest month, minimum temperature in the coldest month, temperature annual range, annual precipitation, precipitation in the wettest month, precipitation in the driest month, and precipitation seasonality (coefficient of variation of monthly precipitation expressed as a percentage; Fick & Hijmans, 2017).

For each bird species, correlated predictors (|*r*| ≥0.75) were removed by selecting the best‐fitting predictor (using Akaike information criterion corrected for small sample size (AICc); Barbosa, 2015). The best predictors from all combinations were determined through the R package enmSdm (Smith, 2017). We tested five regularization parameters for each species: 0.5, 1, 1.5, 2, and 5 (Warren & Seifert, 2011). Overall best predictors and regularization parameters were determined through AICc model selection. The best Maxent model for each bird species was projected for current climate and future climate.

We then evaluated insectivorous bird species at a regional level using individual species distribution models. To estimate richness, each model of continuous suitability was modified using a threshold to create areas of presence and absence. The threshold value was based on the true skill statistic which maximizes the sum of sensitivity (proportion of accurately predicted presences) and specificity (proportion of accurately predicted absences) using a logistic threshold for each bird species (Bean et al., 2012; Liu et al., 2005, 2016). For each bird species, areas that had a suitability over the thresholded value showed the species as present. We examined mean suitability for each avian insectivore species and compared total area of presence for current and future climate models. We determined avian richness over East Africa by adding all thresholded layers together. We obtained *Coffea arabica* and *Coffea canephora (*Robusta*)* locations from GBIF and from the International Center for Tropical Agriculture (*n* = 207) (CIAT; Figure S1; GBIF.org, 2018; Ovalle‐Rivera et al., 2015). By far, most coffee grown in East Africa is Arabica, and though Uganda also produces Robusta, we lacked data to distinguish the two, and as such, they were pooled for analyses. We extracted predicted bird richness for the projected climate scenarios at each coffee farm.

### 2.5 Microclimate assessment

**Figure 3:**
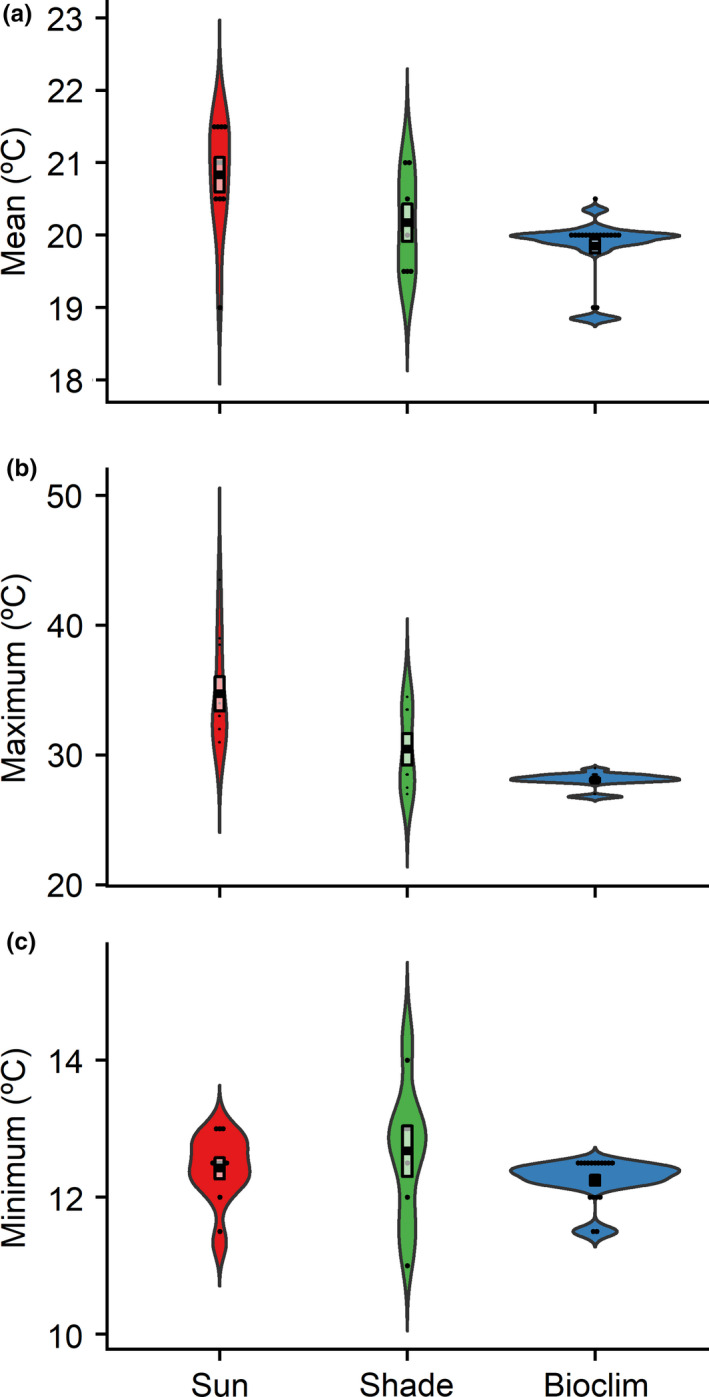
Violin plot comparison of temperatures of iButtons placed in the sun versus iButtons placed in the shade (*n* = 17 from 13 sites) compared with Worldclim layers for mean monthly temperature (a), maximum monthly temperature (b), and minimum monthly temperature (c). The plots demonstrate the probability density of the data smoothed by a kernel density estimator, with the boxes demonstrating the mean ± interquartile range, while dots are iButton temperatures

Finally, to characterize the mitigating impact of shade trees on local scale climate on coffee farms, we first compared mean monthly temperature, maximum monthly temperature, and minimum monthly temperature from the deployed iButtons to the same variables obtained from WorldClim data extracted from site locations (average over 1970–2000) for December, January, February, and March (the months the iButtons were deployed; Fick & Hijmans, 2017). The mean temperature of WorldClim layers for December through March at our site locations was similar to temperatures recorded by our iButton loggers deployed in shade and was less than the temperatures we recorded in sun (see Section 3; Figure 3). Therefore, we simulated climate impacts of the loss of shade trees by adding the difference in maximum temperature of warmest month, minimum temperature of coldest month, and annual mean temperature between sun and shade from the iButtons to temperature Bioclim layers for maximum temperature of warmest month, minimum temperature of coldest month, and annual mean temperature, respectively, for current and future (2075, 8.5 representative concentration pathway (RCP)) climates, thereby creating adjusted Bioclim layers. We were unable to adjust other Bioclim layers due to limited data availability. Models were then reprojected on adjusted Bioclim layers to simulate climatic conditions if shade trees were removed from coffee farms. We then conducted the same calculations of richness, suitability, and suitable area on adjusted climate layers as nonadjusted climate layers.

## 3 RESULTS

Worldclim mean monthly temperature, maximum monthly temperature, and minimum monthly temperature averaged across the sites and months, the iButtons were deployed differed from both sun and shade iButton temperatures (Figure 3, Appendix Table S3). The mean temperature from WorldClim was 0.98º C cooler than mean sun temperature on sites (Welch two‐sample one‐tailed *t* test, *t* = 3.54, *df* = 13.60, *p* = .002) and 0.32°C cooler than mean shaded temperature on sites (Welch two‐sample one‐tailed *t* test, *t* = 1.22, *df* = 9.98, *p* = .125) (Figure 3, Table S3). The maximum monthly WorldClim temperature was 6.64º C lower than the maximum monthly sun iButton temperature (Welch two‐sample one‐tailed *t* test, t = 4.92, *df* = 9.32, *p* < .001) and 2.37º C lower than the maximum monthly shade iButton temperature (Welch two‐sample one‐tailed *t* test, t = 2.01, *df* = 6.36, *p* = .044) (Figure 3, Appendix Table S3). The monthly minimum WorldClim temperature was 0.17º C lower than the minimum monthly sun iButton temperature (Welch two‐sample one‐tailed *t* test, *t* = 0.97, *df* = 14.65, *p* = .35) and 0.42°C lower than the minimum monthly shade iButton temperature (Welch two‐sample one‐tailed *t* test, *t* = 1.10, *df* = 7.44, *p* = .30) (Figure 3, Table S3).

From the pool of bioclimatic variables used to model species distributions, precipitation in the driest month and precipitation seasonality were selected in the best model for 92.2% of 77 bird species, while minimum temperature in the coldest month was selected in the best model for only 5.2% of bird species (Table 2). The best regularization parameter for all birds was 0.5 (Supplementary Dataset S1). The mean suitability threshold value across the 77 species was 0.41 (standard deviation = 0.03), with a maximum threshold value of 0.48, and a minimum of 0.32.

**Table 2:** Percent of best Maxent models and number of bird species’ (*n* = 77) best Maxent models (selected by AIC_c_ out of all possible combinations of predictors) that included specified climactic predictors to show importance of specific bioclimatic variables

Predictor	Percent of Models	Number of Species
Precipitation driest month	92.2	71
Precipitation seasonality	92.2	71
Temperature annual range	70.1	54
Annual precipitation	67.5	52
Annual mean temperature	54.2	41
Precipitation wettest month	41.5	32
Maximum temperature warmest month	40.3	31
Mean diurnal range	32.5	25
Mean temperature coldest month	5.2	4

**Figure 4:**
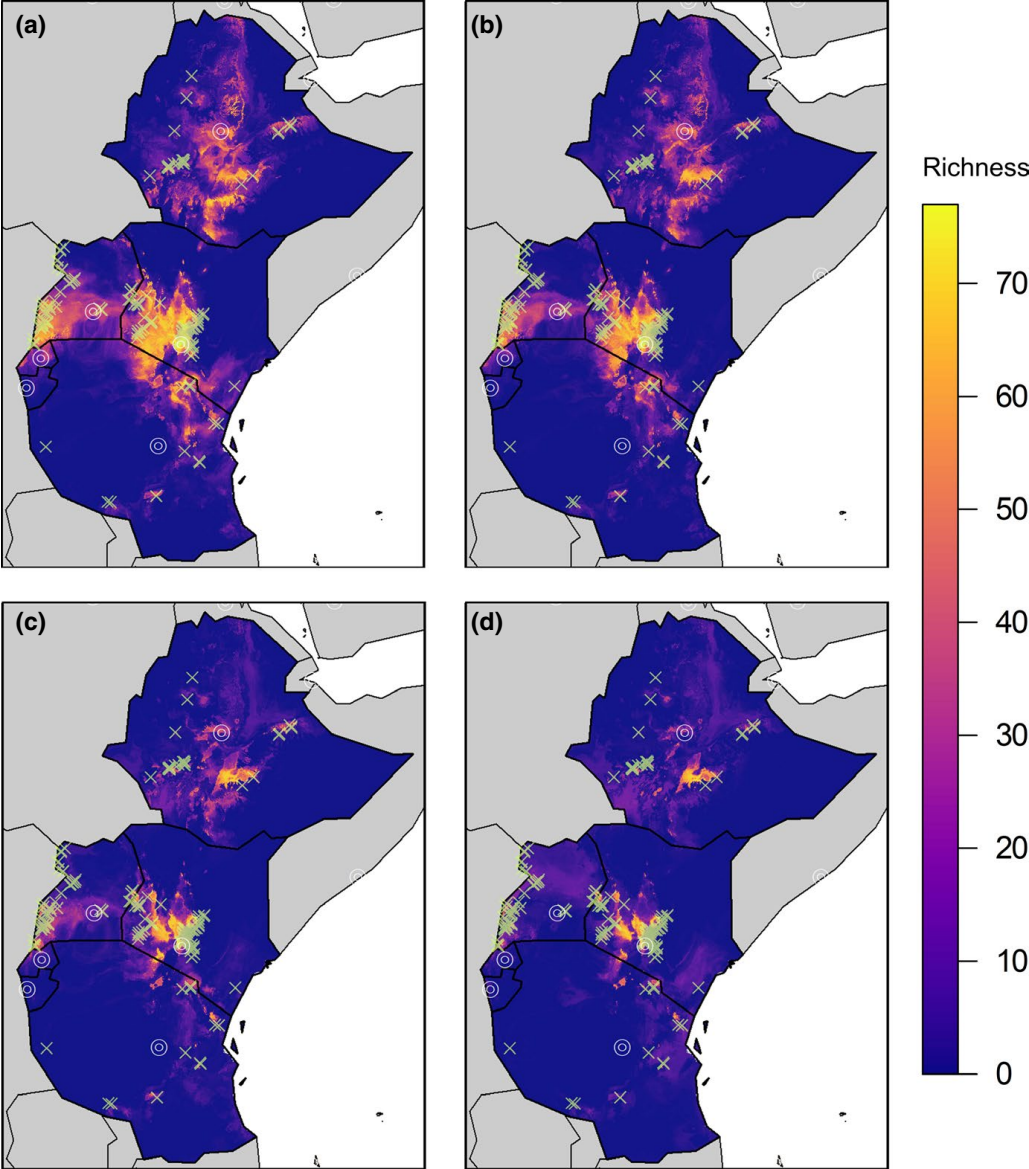
Predicted avian insectivore species richness in East Africa with capitals marked with white circles and locations of coffee farms with transparent green markers using thresholded suitability values for current climate conditions (a), current climate conditions adjusted if shade trees were removed on farms (b), future climate conditions (RCP 8.5, 2075), (c) and future climate conditions (RCP 8.5, 2075) adjusted if shade trees were removed on farms (d)

**Figure 5:**
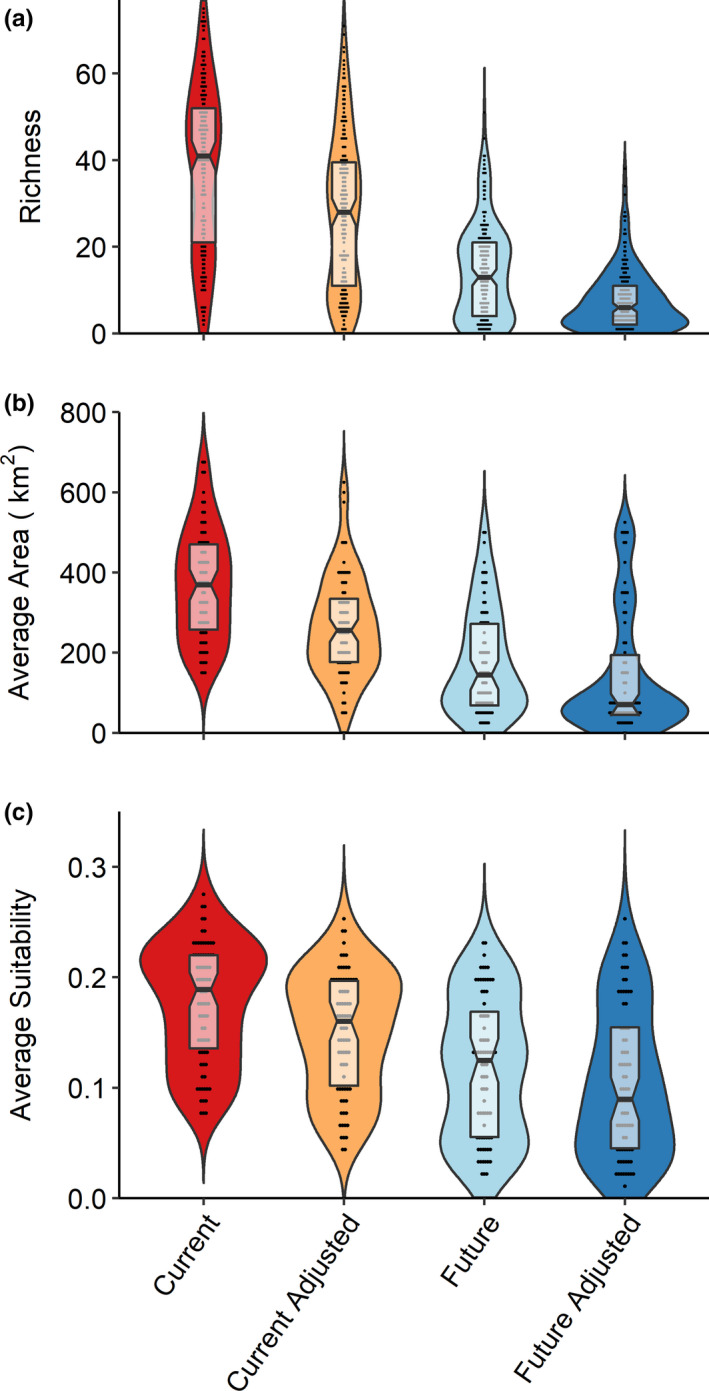
Comparison of projected East African avian insectivore presence (*n* = 77 species) for four climate scenarios (current (red), adjusted (shade tree removal) current (orange), future (RCP 8.5, 2075)(blue), and adjusted (shade tree removal) future (RCP 8.5, 2075) (dark blue)) using three metrics: projected average avian insectivore richness on coffee farms (a), average climatically suitable area (b)(both identified using sensitivity–specificity thresholding) and average suitability for avian insectivores (c). The plots demonstrate the probability density of the data smoothed by a kernel density estimator, with the boxes demonstrating the mean ± interquartile range with notches at confidence intervals, while black bars represent grouped observations (number of coffee farms in a, number of species in b, c)

Avian biodiversity on coffee farms is projected to decrease from a mean of 37.9 insectivorous bird species on East African coffee farms (95% CI 35.9, 40.6) to a mean of 28.7 bird species (95% CI 25.9, 31.4) for a RCP of 2.6 and further to a mean of 14.1 bird species for an RCP of 8.5 (95% CI 12.6, 15.7) (Figures 4, 5). Mean suitability for avian insectivores across East Africa is projected to decrease by 24.3 percent for an RCP of 2.6 and 33.2 percent for an RCP of 8.5 (Figure 5). Similarly, mean area suitable for avian insectivores is projected to decrease by 38.1 percent for an RCP of 2.6 and 62.7 percent for an RCP of 8.5 (calculated through area from thresholded suitability predictions) by 2075 (Figure 5).

We projected climatic changes associated with the removal of all shade trees on coffee farms by adding the differences in temperature in sun and in shade on coffee farms to the corresponding Bioclim layers. We found that given estimated climatic changes if all shade trees were immediately removed, current projected richness of avian insectivore species on coffee farms would decline from an average of 37.9 species to an average of 27.5 species solely based on changes in microclimate temperature (95% CI 25.13, 30.0) (Figures 4, 5). Simulating the removal of all shade trees by 2075 (using a representative concentration pathway (RCP) of 8.5), projected bird species’ richness decreases further to a mean of 7.6 bird species on each farm (CI = 6.6, 8.5) (Figures 4, 5). Similarly, using the 8.5 RCP climatic scenario and simulating the removal of shade trees by 2075 by adjusting temperatures of the Bioclim layers, mean suitable area is projected to decrease by 51.6 percent, and mean suitability is projected to decrease by 33.4 percent (Figure 5).

## 4 DISCUSSION

Our study found that regional impacts of climate change may be mediated locally by the presence of shade. Shade trees on coffee farms have previously been found to increase biodiversity by providing structural habitat (Buechley et al., 2015; Jha et al., 2014; Perfecto et al., 1996; Philpott & Bichier, 2012), and a loss of shade trees has been shown to reduce bird diversity in the Neotropics (Philpott & Bichier, 2012). Our study broadens previous work by demonstrating that shade significantly lowers temperature on coffee farms and therefore contributes to the preservation of avian insectivore diversity. Moreover, higher temperatures bolster insect pest growth and reproduction rates in coffee (Jaramillo et al., 2011), so shade trees may help manage pests both by dampening bottom‐up forces of increased temperature on insect pests and preserving top‐down effects of birds that serve as their natural enemies (Classen et al., 2014; Dainese et al., 2019; Johnson et al., 2010; Maas et al., 2016). Pest‐eating birds in coffee consume far more insects than just the pests (Sherry et al., 2016) and so can be maintained with shade trees even as pest numbers diminish (Railsback & Johnson, 2014). Intraguild predation, by which birds reduce insect enemies of coffee pests (Perfecto et al., 2014), is possible but enclosure studies suggest a net positive effect of birds on pests (Maas et al., 2016). It is clear that shade trees currently buffer extreme temperatures (Garedew et al., 2017), and the future effects of shade removal will become more critical for avian insectivores on coffee farms.

Additionally, this study agrees with previous findings on the impacts of climate change on species richness and climatically suitable area for East African avian insectivores (Moritz & Agudo, 2013; Walther, 2010). Specifically, our findings agree with many other studies establishing that climate change is projected to decrease avian insectivore richness and areas that are climatically suitable for avian insectivores, even without accounting for habitat modification (Jetz et al., 2007; Salas et al., 2017; Tingley et al., 2009).

East Africa will become one of the most suitable areas to produce coffee in the future (Bunn, et al., 2015; Ovalle‐Rivera et al., 2015), and use of shade trees on coffee plantations in East Africa is increasing (Coffee Research Institute, 2019, Schooler, unpublished data). However, coffee plantations, especially at lower elevations, are undergoing widespread conversion to urban and suburban landscapes (Jaramillo et al., 2013), and if this rapid urbanization triggers the removal of shade trees on coffee farms, it will have drastic implications on climate, microclimate, and projected bird richness (Peters et al., 2019; Philpott et al., 2008; Rahn et al., 2014). Should urbanization continue, bird richness declines from habitat loss will be exacerbated by the drastic changes in temperature from reduction of shade trees (Buechley et al., 2015; Peters et al., 2019; Philpott et al., 2008).

Though the benefit of shade coffee on avian richness in East Africa is less resolved than in the Neotropics (Buechley et al., 2015; Classen et al., 2014; Milligan et al., 2016; Smith et al., 2015), all findings agree that there can be fewer pests and increased coffee yield on shaded coffee farms both in the Neo‐ and Paleotropics (Jaramillo et al., 2009; Nesper et al., 2017) and that avian insectivores contribute to pest control (Classen et al., 2014; Dainese et al., 2019; Karp et al., 2014; Sherry et al., 2016). As climate warms, more species will become restricted by climate on a region‐wide level. Shade trees on farms will help to maintain the avian insectivore species pool on both a regional and a local scale through the mitigating effects of shade on temperature. Conversely, shade tree loss will not only cause biodiversity loss due to loss of habitat, but also will exacerbate warming temperatures, causing further declines in richness. Coffee, especially shade coffee, can act in conjunction with forested habitats to maintain bird diversity and pest control services (Buechley et al., 2015; Karanth et al., 2016). To preserve avian insectivore diversity, shade trees must be maintained on coffee farms in East Africa. Future increases in temperature will limit avian insectivore species distributions at the regional level, thus affecting bird distributions on individual coffee farms.

Our finding that monthly Worldclim averages match more closely with iButton temperatures in shade than those iButton temperatures in sun suggests that use of Bioclim layers for species distribution modeling may rely on assumptions about landcover type at any given location. The Bioclim model is interpolated from data collected at weather stations, so it is likely that these weather stations were in shaded areas and thus not capturing the temperatures in full‐sun areas of coffee farms (Fick & Hijmans, 2017). An alternate explanation for the differences we found between the Bioclim temperatures and temperatures on sun coffee farms is global climatic warming. Given that current Bioclim data are an average of temperatures and precipitation from 1970 to 2000 and our sampling was conducted in 2018, it is possible that the iButton data simply demonstrate a clear trend of global climate change congruent with estimated predictions. Yet, based upon current IPCC estimates, the climate in East Africa has been changing by 0.005º C per year (IPCC, 2007). Even if Bioclim temperatures were from 1970, climate change only accounts for a 0.25º C change over 50 years, which our estimates of sun mean temperatures on coffee farms exceed by 376% (6.61 º C over Bioclim). This is a caveat for other research that uses Bioclim data for habitat suitability models, especially in locations of limited availability of high‐resolution climate data. However, an important limitation of this finding is that we were only able to use 13 data points for 3 months given limitations of data collection and that iButtons were unshielded, which may have biased measurements and results (Terando et al., 2017). However, we believe that because we found significant differences between sun and shade, compared Worldclim temperature layers only to the months that we collected data, and deployed iButtons during a time period covering both the wet and the dry seasons, we believe these biases were minimized. Additionally, iButtons were placed under cover of coffee bushes, thus out of direct sunlight, and because we were comparing temperatures from iButtons deployed with the same treatment, the bias is negligible for the temperature adjustment of the Bioclim layers. However, this bias should be taken into consideration when comparing the temperatures we found to temperatures from Bioclim layers.

Precipitation in the driest month, precipitation seasonality, and annual precipitation was selected more than temperature in the best Maxent models for species. This is consistent with some studies which indicate precipitation, especially precipitation extremes, may be more important than temperature in determining climatic suitability (Echeverri et al., 2019; Rapacciuolo et al., 2014; Tingley et al., 2009). Although shade will not have direct effects on precipitation on coffee farms, shade has been linked to higher humidity, which then may mitigate projected future decreases in precipitation due to climate change (Garedew et al., 2017; IPCC, 2007; Mariño et al., 2016; Meylan et al., 2017). Though precipitation was selected in the majority of models over temperature variables, our modifications of temperature layers clearly impacted many insectivorous bird species modeled, indicating that for the species where temperature was selected as a best predictor, even a minor change in temperature could have drastic impacts.

One important limitation of our findings is that any large‐scale species distribution modeling approach has sources of error, especially when using thresholded values to estimate species richness (“stacked species distribution models”) (Benito et al., 2013; Calabrese et al., 2014; Engler et al., 2017). Though other models, especially ensemble models, have been found to perform better when creating stacked species distribution models, many of these models require high computing power and show poor performance when transferring across time or space (Benito et al., 2013; Calabrese et al., 2014). In addition, we used published best practices in Maxent modeling through our selection of predictors, detailed model selection procedure, use of species with greater than 50 data points, and dynamic threshold determination (Araújo et al., 2004; Bean et al., 2012; Liu et al., 2016; Phillips & Dudík, 2008; Warren & Seifert, 2011). However, these important sources of error must be taken into consideration.

Projecting suitability models onto new climactic scenarios also has inherent sources of error (Elith et al., 2010; Yates et al., 2018). For example, we found that reduction of bird diversity in the model for the future climate compared to the future climate with shade trees removed was not as drastic as the reduction of bird diversity in the model for the current climate compared to the current climate with shade trees removed. This is likely because the temperature adjustment led Maxent to extrapolate to novel environmental scenarios, violating the assumption that relevant environmental gradients were adequately sampled (Appendix Figure S2; Elith & Leathwick, 2009; Mesgaran et al., 2014; Yates et al., 2018). Alternatively, only extremely tolerant avian insectivore species remained in the future projection, and there were few of those species that were removed with the more drastically warmed climate in the adjusted future projection.

Additionally, there are inherent sources of error associated with any climate change projection model (Onyutha et al., 2016; Pearson & Dawson, 2003; Seid & Shemelis, 2013), and though we believe we minimized this error through using a model found to be accurate in East Africa (Onyutha et al., 2016; Seid & Shemelis, 2013), there is still the possibility of sources of error from the climatic data. Thus, our future‐adjusted results for an extreme climatic scenario should be viewed with those caveats.

Our results suggest that the projected climatic shift from loss of shade trees has a dramatic effect similar to a conservative climate change emissions scenario. Not only will loss of shade cause increases in temperature as shown by our models, but it will also cause loss of habitat and a disruption of other biotic interactions that we did not model (Philpott et al., 2008; Scherer et al., 2016). This is consistent with evidence that habitat loss in addition to climate change will drastically alter species distributions (Fischer & Lindenmayer, 2007; Intergovernmental Science‐Policy Platform on Biodiversity & Ecosystem Services, 2019; Jaramillo et al., 2013; Jetz et al., 2007). While coffee farmers may face the challenges of decreased suitability for coffee plants due to climate change and increased urbanization, it is critical to ensure continued existence and increased use of shade trees to mitigate climate change and its impact on bird diversity (Bunn, et al., 2015; Njiru, 2016; Pham et al., 2019).

As climate for growing coffee becomes increasingly unsuitable, especially at low elevations, it is critical to maintain shade levels on coffee farms in order to mitigate temperature increases. Mitigating temperature increases on coffee farms is crucial not only for coffee physiology and pest physiology, but also for avian insectivore richness that contributes to pest reduction services (Bunn, et al., 2015; Jaramillo et al., 2011; Perfecto et al., 1996; Philpott & Bichier, 2012). Refining adaptation strategies for coffee farms is important both for coffee growers and for the preservation of avian biodiversity, especially for coffee in East Africa, since it will be one of the most climactically suitable areas in the world for growing coffee in the future (Bunn, et al., 2015; DaMatta et al., 2019; Moat et al., 2017).

## CONFLICT OF INTERESTS

The authors declare no competing interests or conflicts of interest.

## AUTHOR CONTRIBUTION

**Sarah L Schooler:** Conceptualization (equal); Data curation (lead); Formal analysis (lead); Investigation (equal); Methodology (equal); Software (equal); Visualization (lead); Writing‐original draft (lead); Writing‐review & editing (equal). **Matthew D Johnson:** Conceptualization (equal); Funding acquisition (equal); Project administration (lead); Resources (supporting); Supervision (equal); Writing‐review & editing (equal). **Peter Njoroge:** Conceptualization (supporting); Funding acquisition (supporting); Project administration (supporting); Resources (lead); Writing‐review & editing (supporting). **William Tim Bean:** Conceptualization (equal); Data curation (supporting); Formal analysis (supporting); Funding acquisition (equal); Investigation (equal); Methodology (equal); Software (equal); Supervision (equal); Validation (lead); Writing‐review & editing (supporting).

## Supporting information

Supp_InfoS1

Supp_InfoS2

Appendix S1

## Data Availability

The summary temperature data that supports the findings of this study are available Supplementary Dataset S1. All models were generated from openly available datasets from the Global Biodiversity Information Facility at gbif.org (doi and reference numbers not available due to high quantity of datasets used) and Worldclim at worldclim.org (Fick & Hijmans, 2017; GBIF.org, 2018). Selected model parameters for each species are provided in Supplementary Dataset S2, and full models, datasets for each individual species, coffee farm locations, iButton data, and code scripts are available on Dryad (https://doi.org/10.5061/dryad.8931zcrmz).
