# Supplementary material for: Shade trees preserve avian insectivore biodiversity on coffee farms in a warming climate
Source: Ecol Evol. 2020 Oct 23;10(23):12960–72. doi: 10.1002/ece3.6879 (PMC7713971; doi:10.1002/ece3.6879)
Supplement: Supplementary file 3 — Appendix S1 [file ECE3-10-12960-s003.docx]

**Appendices**

Appendix Table S1. Scientific name, common name, guild (identified as G, with omnivores identified as O, and insectivores identified as I), if the bird as migratory (identified as M), family, length in centimeters (identified as L (cm)), and weights in grams (identified as W (g)) for avian insectivores included in analysis in this study (Smith et al. 2015, del Hoyo et al. 2018).

| Scientific Name | Common Name | G | M | Family | L (cm) | W (g) |
| --- | --- | --- | --- | --- | --- | --- |
| *Amblyospiza albifrons* | Thick-billed Weaver | O |  | *Ploceidae* | 18 | 45.5 |
| *Anaplectes rubriceps* | Red-headed Weaver | O |  | *Ploceidae* | 13.5 | 21.5 |
| *Anthus trivialis* | Tree Pipit | I | M | *Motacillidae* | 14.5 | 27 |
| *Apalis cinerea* | Grey Apalis | I |  | *Cisticolidae* | 12.5 | 11 |
| *Apalis flavida* | Yellow-breasted Apalis | I |  | *Cisticolidae* | 11.5 | 8 |
| *Batis molitor* | Chinspot Batis | I |  | *Platysteiridae* | 12 | 9.5 |
| *Bradypterus cinnamomeus* | Cinnamon Bracken Warbler | I |  | *Sylviidae* | 14.5 | 18 |
| *Camaroptera brachyura* | Green-backed Camaroptera | I |  | *Cisticolidae* | 10.5 | 10 |
| *Chalcomitra amethystina* | Amethyst Sunbird | O |  | *Nectariniidae* | 14 | 13.4 |
| *Chalcomitra senegalensis* | Scarlet-chested Sunbird | O |  | *Nectariniidae* | 14 | 12 |
| *Cinnyricinclus leucogaster* | Violet-backed Starling | O | M | *Sturnidae* | 16 | 40 |
| *Cinnyris chalybeus* | Collared Sunbird | O |  | *Nectariniidae* | 10 | 8 |
| *Cinnyris venustus* | Variable Sunbird | O |  | *Nectariniidae* | 10.5 | 7.5 |
| *Cisticola cantans* | Singing Cisticola | I |  | *Cisticolidae* | 13 | 14 |
| *Cisticola chiniana* | Rattling Cisticola | I |  | *Cisticolidae* | 13.5 | 15 |
| *Cisticola erythrops* | Red-Faced Cisticola | I |  | *Cisticolidae* | 14 | 14.5 |
| *Cisticola robustus* | Stout Cisticola | I |  | *Cisticolidae* | 14.5 | 20.5 |
| *Cossypha caffra* | Cape Robin-Chat | I |  | *Muscicapidae* | 16.5 | 30.5 |
| *Cossypha semirufa* | Rüppell's Robin-Chat | I |  | *Muscicapidae* | 18.5 | 26.5 |
| *Crithagra citrinelloides* | African Citril | O |  | *Fringillidae* | 11.5 | 13 |
| *Crithagra mozambica* | Yellow-fronted Canary | O |  | *Fringillidae* | 12 | 14 |
| *Crithagra sulphurata* | Brimstone Canary | O |  | *Fringillidae* | 15 | 20 |
| *Crithagra xanthopygia* | Yellow-rumped Seedeater | O |  | *Fringillidae* | 11 | 12 |
| *Dicrurus adsimilis* | Fork-tailed Drongo | I |  | *Dicruridae* | 25 | 50 |
| *Dryoscopus cubla* | Black-backed Puffback | O |  | *Malaconotidae* | 17 | 27.5 |
| *Emberiza flaviventris* | Golden-breasted Bunting | O |  | *Fringillidae* | 15.5 | 21 |
| *Eminia lepida* | Grey-capped Warbler | I |  | *Cisticolidae* | 15 | 10 |
| *Estrilda astrild* | Common Waxbill | O |  | *Estrildidae* | 11 | 8.5 |
| *Estrilda rhodopyga* | Crimson-rumped Waxbill | O |  | *Estrildidae* | 10 | 8 |
| *Euplectes ardens* | Red-collared Widowbird | O |  | *Ploceidae* | 12.2 | 19 |
| *Euplectes capensis* | Yellow Bishop | O |  | *Ploceidae* | 15 | 21.5 |
| *Iduna natalensis* | African Yellow Warbler | I |  | *Acrocephalidae* | 13 | 13 |
| *Lagonosticta rubricata* | African Firefinch | O |  | *Estrildidae* | 10.5 | 9 |
| *Laniarius major* | Tropical Boubou | I |  | *Malaconotidae* | 23 | 53 |
| *Lanius humeralis* | Northern Fiscal | I |  | *Laniidae* | 22 | 41.5 |
| *Lanius phoenicuroides* | Red-tailed Shrike | I | M | *Laniidae* | 17.5 | 30 |
| *Macronyx croceus* | Yellow-throated Longclaw | I |  | *Motacillidae* | 21 | 50.5 |
| *Melaenornis fischeri* | White-eyed Slaty Flycatcher | I |  | *Muscicapidae* | 15 | 24.5 |
| *Melaenornis microrhynchus* | African Grey Flycatcher | I |  | *Muscicapidae* | 14 | 17.5 |
| *Melaenornis pallidus* | Pale Flycatcher | I |  | *Muscicapidae* | 16 | 22.25 |
| *Monticola saxatilis* | Common Rock Thrush | O | M | *Muscicapidae* | 17 | 50 |
| *Motacilla aguimp* | African Pied Wagtail | I |  | *Motacillidae* | 20 | 27.5 |
| *Motacilla flava* | Western Yellow Wagtail | I | M | *Motacillidae* | 16.5 | 18.5 |
| *Muscicapa striata* | Spotted Flycatcher | I | M | *Muscicapidae* | 14 | 16 |
| *Nectarinia kilimensis* | Bronze Sunbird | O |  | *Nectariniidae* | 17 | 15.45 |
| *Nectarinia tacazze* | Tacazze Sunbird | O |  | *Nectariniidae* | 22 | 15 |
| *Parus albiventris* | White-bellied Tit | I |  | *Paridae* | 14.5 |  |
| *Passer griseus* | Northern Grey-headed Sparrow | O |  | *Passeridae* | 15.5 | 24.8 |
| *Phyllolais pulchella* | Buff-bellied Warbler | I |  | *Cisticolidae* | 10.5 | 7 |
| *Phylloscopus collybita* | Common Chiffchaff | O | M | *Phylloscopidae* | 11.5 | 8.5 |
| *Phylloscopus trochilus* | Willow Warbler | I | M | *Phylloscopidae* | 12 | 10.25 |
| *Plocepasser mahali* | White-browed Sparrow-Weaver | O |  | *Ploceidae* | 17 | 45 |
| *Ploceus baglafecht* | Baglafecht Weaver | O |  | *Ploceidae* | 15 | 30.5 |
| *Ploceus cucullatus* | Village Weaver | O |  | *Ploceidae* | 17 | 36 |
| *Ploceus ocularis* | Spectacled Weaver | I |  | *Ploceidae* | 16 | 27 |
| *Ploceus spekei* | Speke's Weaver | O |  | *Ploceidae* | 15 | 35.5 |
| *Ploceus xanthops* | Holub's Golden Weaver | O |  | *Ploceidae* | 17.5 | 40 |
| *Prinia subflava* | Tawny-flanked Prinia | I |  | *Cisticolidae* | 11.5 | 9 |
| *Pycnonotus barbatus* | Common Bulbul | O |  | *Pycnonotidae* | 17.5 | 35 |
| *Saxicola rubetra* | Whinchat | I | M | *Muscicapidae* | 13 | 19.5 |
| *Saxicola torquatus* | African Stonechat | I | M | *Muscicapidae* | 12.5 | 15 |
| *Sylvia atricapilla* | Eurasian Blackcap | O | M | *Sylviidae* | 14 | 20 |
| *Sylvia communis* | Common Whitethroat | O | M | *Sylviidae* | 14 | 16.5 |
| *Sylvia lugens* | Brown Parisoma | I |  | *Sylviidae* | 13.5 | 15 |
| *Sylvietta whytii* | Red-faced Crombec | I |  | *Macrosphenidae* | 9 | 10 |
| *Tchagra australis* | Brown-crowned Tchagra | I |  | *Malaconotidae* | 18 | 37.5 |
| *Tchagra senegalus* | Black-crowned Tchagra | I |  | *Malaconotidae* | 21 | 47 |
| *Terpsiphone viridis* | African Paradise Flycatcher | I | M | *Monarchidae* | 18 | 13 |
| *Turdoides hindei* | Hinde's Babbler | I |  | *Leiothrichidae* | 21.5 | 67.5 |
| *Turdoides hypoleuca* | Northern Pied Babbler | O |  | *Leiothrichidae* | 23.5 | 73.5 |
| *Turdus olivaceus* | Olive Thrush | O |  | *Turdidae* | 22 | 70 |
| *Uraeginthus bengalus* | Red-cheeked Cordon-bleu | O |  | *Estrildidae* | 12.5 | 10 |
| *Uraeginthus ianthinogaster* | Purple Grenadier | O |  | *Estrildidae* | 13.5 | 14 |
| *Vidua chalybeata* | Village Indigobird | O |  | *Viduidae* | 10.5 | 13 |
| *Vidua macroura* | Pin-tailed Whydah | O |  | *Viduidae* | 11.5 | 14 |
| *Zosterops abyssinicus* | Abyssinian White-eye | O |  | *Zosteropidae* | 10.2 | 10 |
| *Zosterops kikuyuensis* | Kikuyu White-eye | O |  | *Zosteropidae* | 11.5 | 12 |

Appendix Table S2. Scientific name, common name and number of GBIF observations (GBIF Obs.) for avian insectivores included in analysis in this study (n = 77).

| Scientific Name | Common Name | GBIF Obs. |
| --- | --- | --- |
| *Amblyospiza albifrons* | Thick-billed Weaver | 6056 |
| *Anaplectes rubriceps* | Red-headed Weaver | 4580 |
| *Anthus trivialis* | Tree Pipit | 1984 |
| *Apalis cinerea* | Grey Apalis | 2382 |
| *Apalis flavida* | Yellow-breasted Apalis | 9846 |
| *Batis molitor* | Chinspot Batis | 10400 |
| *Bradypterus cinnamomeus* | Cinnamon Bracken Warbler | 8468 |
| *Camaroptera brachyura* | Green-backed Camaroptera | 482 |
| *Chalcomitra amethystina* | Amethyst Sunbird | 2504 |
| *Chalcomitra senegalensis* | Scarlet-chested Sunbird | 19888 |
| *Cinnyricinclus leucogaster* | Violet-backed Starling | 8042 |
| *Cinnyris chalybeus* | Collared Sunbird | 13796 |
| *Cinnyris venustus* | Variable Sunbird | 5470 |
| *Cisticola cantans* | Singing Cisticola | 19676 |
| *Cisticola chiniana* | Rattling Cisticola | 6384 |
| *Cisticola erythrops* | Red-Faced Cisticola | 11356 |
| *Cisticola robustus* | Stout Cisticola | 4698 |
| *Cossypha caffra* | Cape Robin-Chat | 2768 |
| *Cossypha semirufa* | Rüppell's Robin-Chat | 7420 |
| *Crithagra citrinelloides* | African Citril | 7312 |
| *Crithagra mozambica* | Yellow-fronted Canary | 6190 |
| *Crithagra sulphurata* | Brimstone Canary | 4450 |
| *Crithagra xanthopygia* | Yellow-rumped Seedeater | 270 |
| *Dicrurus adsimilis* | Fork-tailed Drongo | 22724 |
| *Dryoscopus cubla* | Black-backed Puffback | 9540 |
| *Emberiza flaviventris* | Golden-breasted Bunting | 3778 |
| *Eminia lepida* | Grey-capped Warbler | 6524 |
| *Estrilda astrild* | Common Waxbill | 7080 |
| *Estrilda rhodopyga* | Crimson-rumped Waxbill | 3564 |
| *Euplectes ardens* | Red-collared Widowbird | 3386 |
| *Euplectes capensis* | Yellow Bishop | 5634 |
| *Iduna natalensis* | African Yellow Warbler | 11274 |
| *Lagonosticta rubricata* | African Firefinch | 2844 |
| *Laniarius major* | Tropical Boubou | 3410 |
| *Lanius humeralis* | Northern Fiscal | 5152 |
| *Lanius phoenicuroides* | Red-tailed Shrike | 1716 |
| *Macronyx croceus* | Yellow-throated Longclaw | 962 |
| *Melaenornis fischeri* | White-eyed Slaty Flycatcher | 7798 |
| *Melaenornis microrhynchus* | African Grey Flycatcher | 7798 |
| *Melaenornis pallidus* | Pale Flycatcher | 12502 |
| *Monticola saxatilis* | Common Rock Thrush | 2202 |
| *Motacilla aguimp* | African Pied Wagtail | 25366 |
| *Motacilla flava* | Western Yellow Wagtail | 9928 |
| *Muscicapa striata* | Spotted Flycatcher | 5124 |
| *Nectarinia kilimensis* | Bronze Sunbird | 13668 |
| *Nectarinia tacazze* | Tacazze Sunbird | 3662 |
| *Parus albiventris* | White-bellied Tit | 5998 |
| *Passer griseus* | Northern Grey-headed Sparrow | 11938 |
| *Phyllolais pulchella* | Buff-bellied Warbler | 3702 |
| *Phylloscopus collybita* | Common Chiffchaff | 796 |
| *Phylloscopus trochilus* | Willow Warbler | 8172 |
| *Plocepasser mahali* | White-browed Sparrow-Weaver | 14586 |
| *Ploceus baglafecht* | Baglafecht Weaver | 23572 |
| *Ploceus cucullatus* | Village Weaver | 11958 |
| *Ploceus ocularis* | Spectacled Weaver | 7778 |
| *Ploceus spekei* | Speke's Weaver | 6670 |
| *Ploceus xanthops* | Holub's Golden Weaver | 5310 |
| *Prinia subflava* | Tawny-flanked Prinia | 13518 |
| *Pycnonotus barbatus* | Common Bulbul | 45716 |
| *Saxicola rubetra* | Whinchat | 2364 |
| *Saxicola torquatus* | African Stonechat | 5760 |
| *Sylvia atricapilla* | Eurasian Blackcap | 7032 |
| *Sylvia communis* | Common Whitethroat | 2798 |
| *Sylvia lugens* | Brown Parisoma | 1316 |
| *Sylvietta whytii* | Red-faced Crombec | 1068 |
| *Tchagra australis* | Brown-crowned Tchagra | 6684 |
| *Tchagra senegalus* | Black-crowned Tchagra | 7020 |
| *Terpsiphone viridis* | African Paradise Flycatcher | 5864 |
| *Turdoides hindei* | Hinde's Babbler | 19962 |
| *Turdoides hypoleuca* | Northern Pied Babbler | 380 |
| *Turdus olivaceus* | Olive Thrush | 2540 |
| *Uraeginthus bengalus* | Red-cheeked Cordon-bleu | 3046 |
| *Uraeginthus ianthinogaster* | Purple Grenadier | 16734 |
| *Vidua chalybeata* | Village Indigobird | 1912 |
| *Vidua macroura* | Pin-tailed Whydah | 4362 |
| *Zosterops abyssinicus* | Abyssinian White-eye | 10738 |
| *Zosterops kikuyuensis* | Kikuyu White-eye | 3790 |

Appendix Table S3. Extracted temperature values (°C) and 95% confidence intervals from data recorded by 16 iButtons placed under shade trees (n = 7) and in sun (n = 9) with corresponding WorldClim temperature values from 13 sites.

| Value | Shade | | Sun | | WorldClim | |
| --- | --- | --- | --- | --- | --- | --- |
|  | Mean | 95% CI | Mean | 95% CI | Mean | 95% CI |
| Mean Monthly | 20.17 | 19.53, 20.80 | 20.83 | 20.29, 21.37 | 19.85 | 19.65, 20.05 |
| Maximum Monthly | 30.45 | 27.50, 33.40 | 34.72 | 31.72, 37.72 | 28.08 | 27.80, 28.36 |
| Minimum Monthly | 12.67 | 11.77, 13.57 | 12.42 | 12.07, 12.76 | 12.25 | 12.09, 12.40 |
| Mean Overall | 20.03 | 19.50, 20.56 | 20.79 | 20.28, 21.31 |  |  |
| Maximum Overall | 34.57 | 30.51, 38.63 | 38.70 | 35.90, 41.50 |  |  |
| Minimum Overall | 8.93 | 7.41,10.45 | 8.55 | 7.71, 9.38 |  |  |
| Daily Range | 21.67 | 18.17, 25.16 | 26.21 | 23.40, 29.02 |  |  |

Appendix Figure S1. Locations of coffee farm points in East Africa.

Appendix Figure S2. Predicted avian insectivore species’ richness in East Africa using thresholded suitability values for future climate conditions (A) and future climate conditions adjusted if shade trees were removed on farms (B), with areas that were outside the maximum of the current average temperature marked in dark grey and country capitals marked with white circles.
